# Long Noncoding RNAs in Aortic Dissection: Mechanistic Roles and Therapeutic Potential

**DOI:** 10.1002/agm2.70106

**Published:** 2026-08-24

**Authors:** Chao Chang, Yongrong Lan, Zhenhua Wu, Jie Geng, Zhigang Guo, Qingliang Chen

**Affiliations:** ^1^ Department of Cardiovascular Surgery Chest Hospital, Tianjin University Tianjin China; ^2^ Department of Cardiovascular Surgery Fuzhou University Affiliated Provincial Hospital Fuzhou China; ^3^ Department of Cardiovascular Surgery, Fujian Provincial Clinical College of Fujian Medical University Fujian Provincial Hospital Fuzhou China; ^4^ Department of Cardiovascular Surgery, ICU Chest Hospital, Tianjin University Tianjin China; ^5^ Department of Cardiology Chest Hospital, Tianjin University Tianjin China

**Keywords:** aorta, aortic dissection, extracellular matrix, lncRNA, vascular smooth muscle cell

## Abstract

Aortic dissection (AD) is a life‐threatening vascular disorder characterized by separation of the layers of the aortic wall, primarily triggered by an intimal tear or an intramural hemorrhage. Age is a well‐recognized and critical risk factor for AD. Epidemiological evidence consistently demonstrates a strong positive correlation between age and AD incidence, with peak occurrence in the 50–70‐year age group. Its pathogenesis involves vascular inflammation, extracellular matrix (ECM) degradation, vascular smooth muscle cell (VSMC) phenotypic switching, apoptosis, and immune cell infiltration. Two major pathophysiological theories have been proposed: one suggests a primary intimal tear, and the other involves medial degeneration from stress concentration. Long noncoding RNAs (lncRNAs) are transcripts longer than 200 nucleotides and account for ~80% of the human noncoding transcriptome. They exert diverse biological functions (e.g., miRNA sponging, protein scaffolding, transcriptional regulation) and are pivotal in diseases such as cancer, rheumatoid arthritis, and AD. Despite emerging evidence linking lncRNAs to AD, most studies to date have focused only on their role as competing endogenous RNAs (ceRNAs), with limited mechanistic depth and scarce clinical validation. This review summarizes the pathophysiology of AD and systematically discusses lncRNA‐mediated mechanisms in AD progression. We highlight current knowledge gaps and propose future directions for exploring lncRNAs as potential therapeutic targets to improve AD prognosis.

## Graphical abstract

Aortic dissection (AD) involves vascular inflammation, VSMC dysfunction, and ECM degradation. LncRNAs regulate AD progression via ceRNA networks, with great potential as diagnostic biomarkers and therapeutic targets, while further clinical validation is needed.

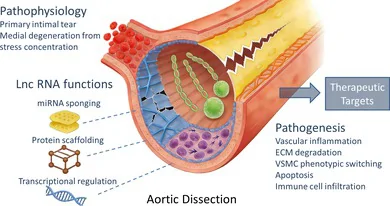

## 1 Overview of Aortic Dissection

Aortic dissection (AD) is a catastrophic vascular disease characterized by a tear in the aortic intima and the formation of true and false lumens, posing a severe threat to patients' lives and health, especially among the elderly. A population‐based study (1995–2015) confirmed that the age‐adjusted incidence of AD rises progressively with advancing age, reaching 4.4 per 100,000 person‐years and has remained stable over decades despite declining trends in other cardiovascular diseases [1].

Elderly AD patients (≥ 65 years) exhibit unique clinical characteristics and greater disease severity. They tend to have more comorbid conditions (e.g., hyperglycemia and diabetes) and are more likely to develop neurological deficits. They also often have fewer typical symptoms (such as abrupt chest or abdominal pain) and lower admission systolic blood pressure, which may mask the diagnosis. Anatomically, elderly patients more frequently suffer DeBakey Type I AD (a severe form involving the ascending aorta), whereas younger patients predominantly present with Type III AD [2]. Patients ≥ 80 years old have a poorer prognosis, including longer hospital stays and nearly double the in‐hospital mortality. Moreover, age ≥ 70 is an independent predictor of in‐hospital death in acute Type A AD (TAAD). The reduced likelihood of surgical intervention in older patients further worsens outcomes [3].

The mechanisms underlying the development and progression of AD are complex. Numerous risk factors (such as chronic arterial hypertension, smoking, and dyslipidemia) can induce aortic degeneration, increase aortic wall fragility, and ultimately lead to an intimal tear and the development of AD [4]. However, the exact pathogenesis remains unclear. Therefore, further in‐depth exploration of the molecular mechanisms of AD and the development of more effective early prevention strategies and targeted therapies is of great significance.

### 1.1 Structure of the Aortic Wall

**FIGURE 1:**
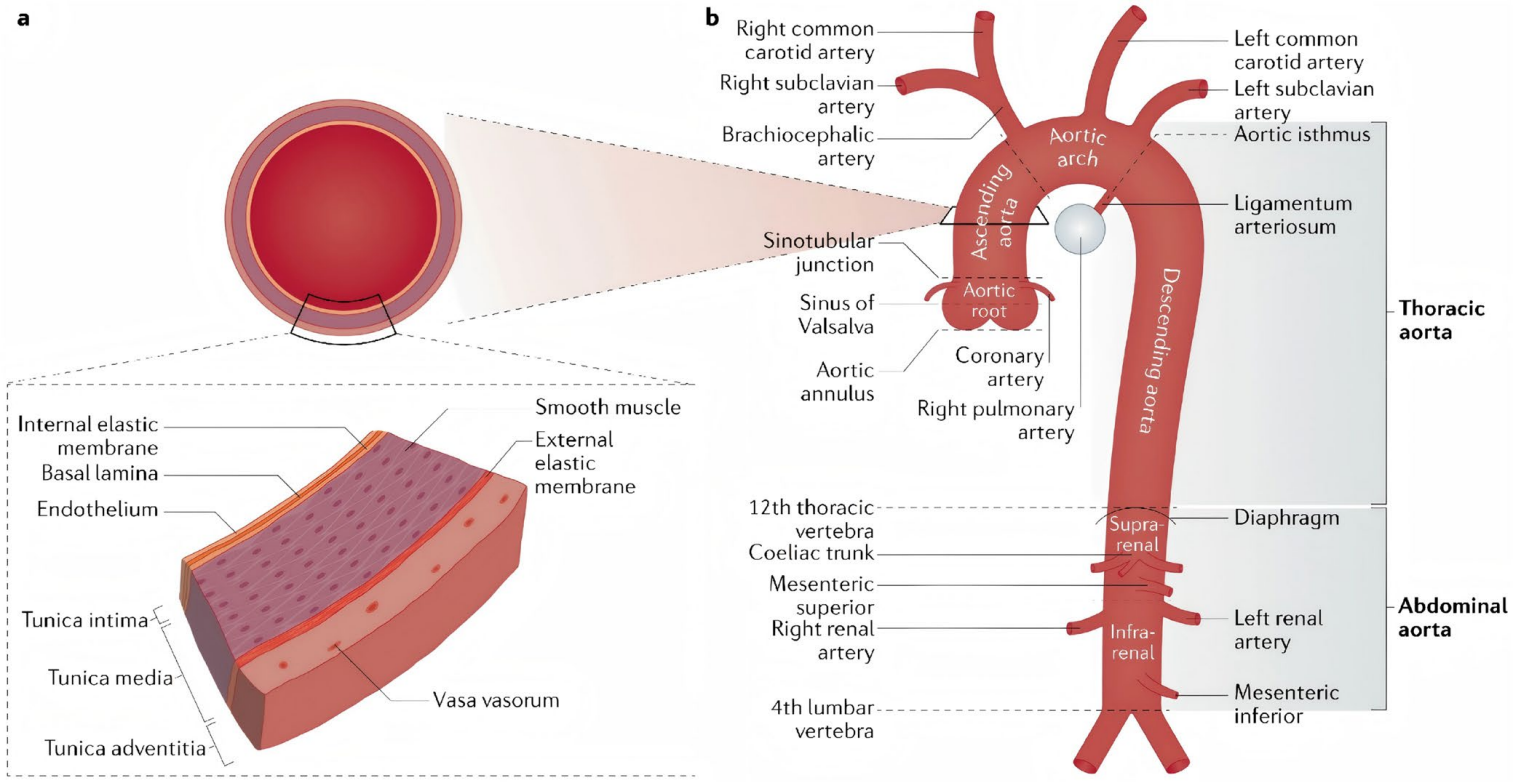
Structure and anatomy of the aorta [5].

The aorta, the largest elastic artery in humans, has three histological layers: intima, media, and adventitia (Figure 1a) [5]. The intima is a single endothelial layer on a Type IV collagen and laminin internal elastic lamina; it acts as a permeability barrier, maintains thrombus resistance, regulates vascular tone via prostacyclin and endothelium‐derived relaxing factor, and participates in inflammation [6]. The adventitia, rich in Type I collagen (the “strength layer”), provides immune surveillance and helps prevent rupture under extreme loads [6, 7]. Comprising 80%–90% of wall thickness, the media absorbs the hemodynamic impact of cardiac contraction. It is composed of collagen, elastic fibers, VSMCs, and ECM, forming tension‐stable concentric lamellar units under normal conditions. The media has “immune privilege”, as its dense elastic layers restrict pathogen entry, immune cell infiltration, and macromolecular diffusion in the wall. VSMCs in the media regulate ECM synthesis and degradation for optimal mechanical function, control blood flow via contraction and relaxation, and adapt to mechanical stress through phenotypic changes, thereby ensuring aortic wall homeostasis [8, 9].

Anatomically, the aorta is divided into the thoracic (root, ascending, arch with branches, descending) and abdominal (suprarenal, infrarenal) segments at the diaphragm, and it bifurcates into the common iliac arteries at the L4 level (Figure 1b) [5, 10, 11, 12].

### 1.2 Pathological Features of Aortic Dissection

AD is defined as a separation of aortic wall layers caused by an intimal rupture or intramural hemorrhage, and is pathophysiologically rooted in medial weakening [13, 14]. Two predominant theories explain AD initiation: (1) a primary intimal tear allows blood to enter the wall, forming an intramural hematoma that dissects the media into true and false lumens [15]; (2) medial stress concentration due to glycosaminoglycan and proteoglycan accumulation alters ECM mechanics—a hallmark of thoracic AD degeneration [16]. AD is also associated with autosomal dominant connective tissue disorders (e.g., Marfan syndrome, vascular Ehlers‐Danlos syndrome, familial thoracic aortic aneurysm and dissection, bicuspid aortic valve) that increase dissection susceptibility by causing aortic structural abnormalities [13, 17].

### 1.3 Pathogenesis of Aortic Dissection

AD pathogenesis involves vascular inflammation, extracellular matrix degradation, VSMC phenotypic transformation and apoptosis, as well as immune cell infiltration. Key mechanisms include inflammation, immune cell infiltration, and VSMC phenotypic changes; however, the exact sequence of events remains unclear due to complex interactions between cellular components and the ECM [18, 19].

#### 1.3.1 Vascular Inflammation, Immune Cell Infiltration and Aortic Dissection

Vascular inflammation drives AD progression: escalating inflammation enhances ECM degradation and VSMC apoptosis, with immune cells (lymphocytes, macrophages, mast cells, neutrophils) infiltrating the aortic wall to mediate damage and remodeling [20, 21]. Elevated IL‐1β in AD samples upregulates key ECM‐degrading enzymes MMP‐2 and MMP‐9, leading to aortic matrix degradation and inducing VSMC apoptosis [22, 23]. Infiltrating immune cells secrete MMPs, adhesion molecules, and reactive oxygen species, thereby altering the microenvironment and promoting VSMC apoptosis [24, 25, 26]. Additionally, MAPK/MMP‐9 signaling, NLRP3–caspase‐1 activation, and neutrophil‐derived factors further amplify inflammation and accelerate AD progression [27, 28, 29, 30].

#### 1.3.2 VSMC Phenotypic Transformation and Aortic Dissection

VSMC phenotypic transformation (from a contractile to a synthetic phenotype) is regulated by factors such as SRF, KLF4, FOXO4, lncRNAs, miRNAs, and TGF‐β through various signal transduction, transcriptional, and epigenetic mechanisms. This phenotypic switching is crucial in the pathogenesis of AD [31, 32]. For example, ACE2 deficiency inhibits inflammation and VSMC phenotypic switching via SIRT3, providing protection against AD [33]. MiR‐145—which is highly expressed in normal aortas but downregulated in AD—helps maintain the VSMC contractile phenotype under mechanical stretch by preventing the downregulation of contractile proteins and phenotypic switching, indicating a regulatory role in AD‐related VSMC plasticity [34]. Aging exacerbates AD through a p53/miR‐1204/MYLK axis that promotes a senescent secretory phenotype in VSMCs and leads to aortic wall damage [35].

#### 1.3.3 VSMC Apoptosis and Aortic Dissection

Forms of cell death include apoptosis, autophagy, pyroptosis, and ferroptosis, and dysregulation of apoptosis is a hallmark of many disorders—including AD [36]. In AD, elevated HCMV‐miR‐US33‐5p promotes aortic VSMC apoptosis by targeting the EPAS1/SLC3A2 pathway [37]. Sirt3 is downregulated in aortic tissues of TAD mice; Sirt3 deficiency increases TAD incidence, whereas Sirt3 overexpression reduces Ang II‐induced ROS production, NF‐κB activation, and VSMC apoptosis [26]. Del‐1 (also known as EDIL3) enhances the clearance of apoptotic VSMCs, helping prevent AD [38]. In AD aortas, miR‐22 is decreased—it normally inhibits VSMC apoptosis via p38 MAPKα, so its downregulation promotes apoptosis (an effect reversible by p38 MAPKα inhibition) [39]. MiR‐4787‐5p is upregulated and induces VSMC apoptosis via the PI3K/Akt/FKHR pathway, further confirming the key role of VSMC apoptosis in AD [40].

## 2 The Influence of LncRNA on the Progression of Aortic Dissection

### 2.1 Overview of LncRNA

Noncoding RNAs (ncRNAs) include miRNAs, lncRNAs, and circRNAs. LncRNAs, accounting for ~80% of the human noncoding transcriptome, regulate gene expression via epigenetic, transcriptional, and posttranscriptional mechanisms [41, 42, 43, 44]. Their expression is dynamically regulated across different cell types, diseases, and developmental stages, and they participate in nearly all steps of gene expression and translation [45, 46].

#### 2.1.1 Structure and Classification of LncRNA

**FIGURE 2:**
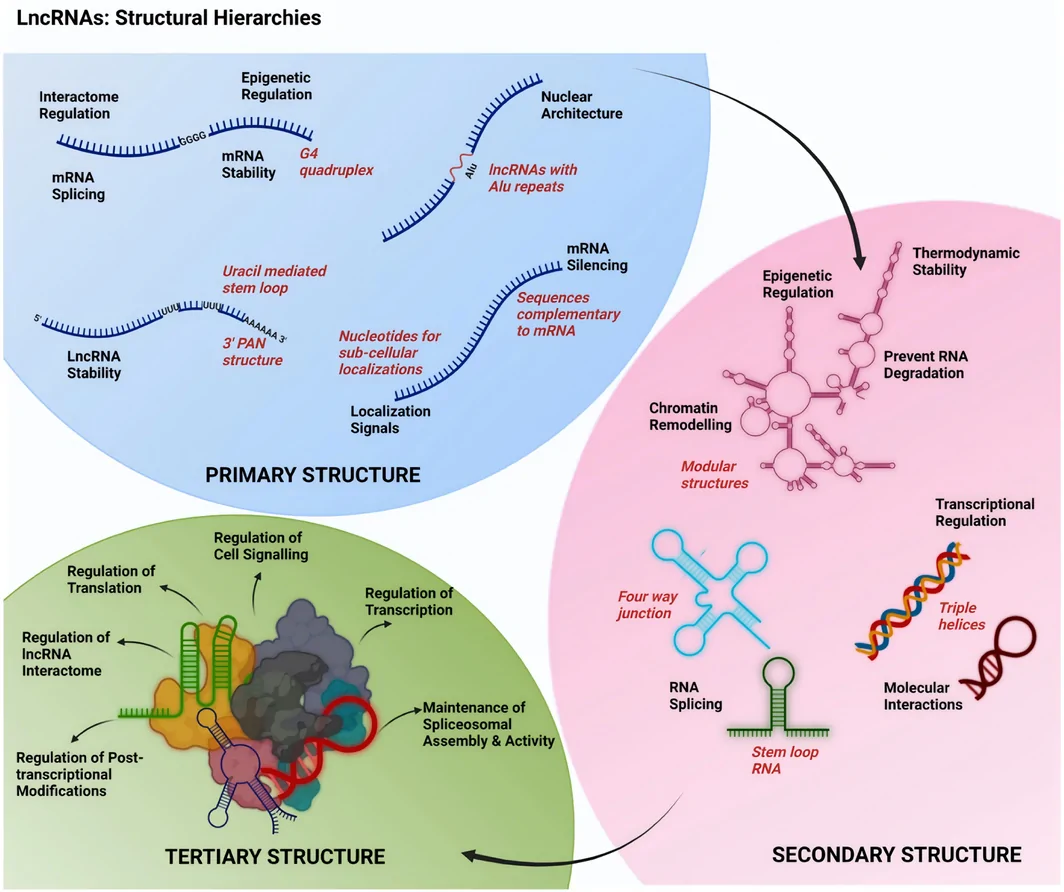
Hierarchical structures of lncRNAs [56].

LncRNAs are noncoding RNA transcripts longer than 200 nt, mostly lacking open reading frames and not translated, with a small subset encoding micropeptides [47]. They are classified by genomic location: sense (overlapping a protein‐coding gene on the sense strand), antisense (overlapping on the antisense strand), bidirectional (sharing a promoter with a coding gene but transcribed in the opposite direction), intronic (derived entirely from an intron), and intergenic (located in regions not overlapping protein‐coding genes) [48, 49, 50]. As of 2022, GENCODE v35 annotates 19,095 lncRNA genes and 54,291 transcripts [51]; LNCipedia v5.2 lists 56,946 lncRNA genes and 127,802 transcripts [52]; NONCODE v5 catalogs 96,308 lncRNA genes and 172,216 transcripts [53]. Similar to mRNAs, most lncRNAs are transcribed by RNA polymerase II and possess typical mRNA features such as 5′‐capping and 3′‐polyadenylation [54]. LncRNAs can adopt complex higher‐order structures, including highly compact tertiary cores (similar to those in ribozymes), dispersed protein‐binding scaffolds, or largely unstructured forms with long disordered regions [55]. Their higher‐order structures may feature motifs such as minor‐groove triplexes, A‐minor interactions, four‐way junctions, kissing loops, pseudoknots, kinks, hooks, ribose zippers, and T‐loops (Figure 2) [56, 57]. A prominent example is the human lncRNA MEG3, which contains multiple tertiary structural elements such as a GNRA tetraloop, a putative T‐loop, and several bulges [58].

#### 2.1.2 Functions of LncRNA

Research into the roles of lncRNAs in disease has burgeoned in recent years. The functions of lncRNAs can be broadly categorized into two classes: cis‐acting and transacting lncRNAs [59, 60]. Cis‐acting lncRNAs modulate the expression of neighboring genes or alter local chromatin state, whereas trans‐acting lncRNAs exert their effects on distant genes throughout the cell. LncRNAs employ diverse mechanisms to execute their functions—for example, they may serve as scaffolds for protein complexes or act as sponges for miRNAs. Through such protein or RNA interactions, lncRNAs regulate a wide spectrum of cellular processes at epigenetic, transcriptional, and posttranscriptional levels.

For instance, the lncRNA SNHG1 stabilizes SERPINA3 mRNA by recruiting the protein HNRNPD, and the resulting upregulation of SERPINA3 promotes colorectal cancer cell migration and invasion [61]. The lncRNA SIMALR promotes nasopharyngeal carcinoma progression by mediating the phosphorylation and activation of eEF1A2, which in turn accelerates the translation of integrin β4 and α6 subunits [62]. LncRNAs can also influence chromatin modifications by interacting with histone regulators (methyltransferases, demethylases, or acetyltransferases). For example, HOTAIR binds to PRC2, leading to repressive H3K27me3 histone methylation. In breast cancer, HOTAIR is overexpressed and collaborates with PRC2 to silence tumor suppressor genes, thereby promoting invasion and metastasis [63, 64, 65]. Beyond protein interactions, lncRNAs frequently act as ceRNAs by binding to miRNAs. In esophageal cancer, LINC00680 sponges miR‐423‐5p, which results in upregulation of the miR‐423‐5p target PAK6, promoting tumor growth [66]. In non‐alcoholic steatohepatitis (NASH), overexpression of lncRNA MIR22HG enhances autophagy and inhibits pyroptosis and fibrosis via the miR‐9‐3p/IGF1 pathway, thereby attenuating NASH progression [67]. In papillary thyroid carcinoma, lncRNA MIAT sponges miR‐150, relieving the repression on its target EZH2 and thus promoting cancer cell invasiveness [68].

In summary, lncRNAs have multifaceted biological functions. Both cis‐ and trans‐acting lncRNAs can regulate gene transcription (locally or at a distance) and interact with proteins and other RNAs, thereby influencing the pathogenesis of various diseases (as exemplified in cancers and NASH). The following sections elaborate on the roles of lncRNAs in AD and summarize the current research progress in this field.

### 2.2 LncRNA and Aortic Dissection

LncRNAs are characterized by their long sequences, intricate three‐dimensional structures, and specific functional domains. They possess multiple binding sites that enable interactions with diverse macromolecules, including DNA, RNA, and proteins. These structural features endow lncRNAs with versatile biological functions, such as regulating gene transcription and acting as RNA sponges. Consequently, lncRNAs play pivotal roles in the development of various cell types, the pathogenesis of different diseases, and the progression of distinct biological stages across organisms. Accumulating evidence from numerous studies has demonstrated that lncRNAs can modulate the progression of AD.

#### 2.2.1 LncRNA H19

LncRNA H19 was one of the first identified nonprotein‐coding RNA molecules. Mounting evidence indicates that lncRNA H19 is implicated in the pathophysiological processes of cardiovascular diseases, emerging as a potential key regulator in various cardiac disorders [69].

Notably, lncRNA H19 is aberrantly overexpressed in the thoracic aortic tissues of patients with AD. It acts as a sponge for miR‐193b‐3p. In vitro, silencing lncRNA H19 in PDGF‐BB‐induced human aortic smooth muscle cells (HASMCs) was found to reverse the regulatory effects of PDGF‐BB on these cells. More interestingly, inhibition of miR‐193b‐3p partially abrogated the effects of H19 silencing. In animal experiments, silencing lncRNA H19 and upregulating miR‐193b‐3p significantly alleviated pathological damage in the thoracic aortas of AD mice. These findings collectively indicate that lncRNA H19 contributes to the initiation and progression of AD [70].

Furthermore, a micropeptide encoded by lncRNA H19—termed glucose metabolism‐regulating small protein (GMRSP) – can suppress hnRNPA2/B1‐mediated alternative splicing of pyruvate kinase M (PKM) pre‐mRNA. This suppression leads to reduced production of PKM2 and impaired glycolysis. Such metabolic reprogramming preserves the contractile phenotype of VSMCs and prevents their phenotypic switch to a proliferative state. These results suggest that GMRSP is a critical regulator of VSMC metabolism and phenotypic stability, highlighting its potential as a therapeutic target for AD [71].

#### 2.2.2 LncRNA XIST

LncRNA XIST plays a crucial role in the pathogenesis of TAAD. XIST is significantly upregulated in the aortic wall tissues of patients with TAAD, and its expression level is closely correlated with clinical prognosis. Functional assays demonstrate that XIST silencing promotes the proliferation and suppresses the apoptosis of VSMCs, whereas restoration of XIST expression has the opposite effects. Mechanistically, XIST harbors binding sites for miR‐17. Downregulation of miR‐17 reverses the enhanced cell proliferation and reduced apoptosis caused by XIST silencing. Further investigations revealed that XIST positively regulates PTEN expression by sponging miR‐17. Collectively, these findings suggest that knockdown of XIST may alleviate the progression of TAAD by activating miR‐17 and modulating downstream PTEN signaling, thereby providing a novel therapeutic target for TAAD management [72].

#### 2.2.3 LncRNA HCG18

LncRNA HCG18 (HLA complex group 18) is located within the human leukocyte antigen complex. Emerging evidence indicates that HCG18 suppresses the proliferation and induces the apoptosis of VSMCs, suggesting a potential involvement in AD pathogenesis [73]. One study found that HCG18 and high mobility group protein A2 (HMGA2) were upregulated, while miR‐103a‐3p was downregulated, in the aortic tissues of patients with AD. Downregulation of HCG18 or upregulation of miR‐103a‐3p enhanced VSMC proliferation and inhibited VSMC apoptosis. Mechanistically, HCG18 promoted HMGA2 expression by competing with miR‐103a‐3p; restoration of HMGA2 expression abrogated the effects of HCG18 downregulation or miR‐103a‐3p upregulation on VSMC proliferation and apoptosis. Additionally, HCG18 knockdown alleviated aortic pathological damage in an AD rat model. Overall, these results suggest that HCG18 impairs VSMC proliferation and induces VSMC apoptosis via the miR‐103a‐3p/HMGA2 axis, thereby exacerbating AD [74].

#### 2.2.4 Lnc‐C2orf63–4‐1

Zhang et al. demonstrated that lnc‐C2orf63‐4‐1 was downregulated in aortic samples from AD patients and in VSMCs challenged with Ang II. This downregulation was correlated with clinical aortic expansion. Moreover, overexpression of lnc‐C2orf63‐4‐1 significantly attenuated Ang II‐induced VSMC apoptosis, phenotypic switching, and ECM degradation both in vitro and in vivo. A customized transcription factor array revealed that signal transducer and activator of transcription 3 (STAT3) served as the key downstream effector. Mechanistically, dual‐luciferase reporter and RNA antisense purification assays showed that lnc‐C2orf63‐4‐1 directly downregulates STAT3 by reducing the stability of STAT3 mRNA. Importantly, upregulation of STAT3 effectively reversed the protective effects of lnc‐C2orf63‐4‐1 against Ang II‐induced vascular remodeling. Therefore, lnc‐C2orf63‐4‐1 negatively regulates STAT3 expression and inhibits the development and progression of AD. This study highlights the crucial role of lnc‐C2orf63‐4‐1 in maintaining vascular homeostasis, as its dysfunction exacerbates Ang II‐induced pathological remodeling [75].

#### 2.2.5 LncRNA OIP5‐AS1

Wang et al. found that LncRNA OIP5‐AS1 was upregulated in Ang II‐treated cells and AD tissues, whereas miR‐143‐3p was downregulated. LncRNA OIP5‐AS1 acts as a ceRNA for miR‐143‐3p, which results in suppressed proliferation and migration but increased apoptosis of human aortic endothelial cells (HAECs) and HASMCs. Concomitantly, OIP5‐AS1 overexpression induced an imbalance between MMP‐2/9 and TIMP‐2/1 in HASMCs, and triggered excessive secretion of IL‐6, IL‐1β, and IL‐17A by human aortic adventitial fibroblasts (HAAFs). Furthermore, manipulating the expression of tubulin (a direct target of miR‐143‐3p) negated the effects of miR‐143‐3p and OIP5‐AS1 on cellular behavior: overexpression or silencing of tubulin effectively abrogated the effects of miR‐143‐3p mimic or OIP5‐AS1, respectively. Overall, these findings indicate that OIP5‐AS1 exacerbates intimal, medial, and adventitial injury of the aorta during AD progression by sponging miR‐143‐3p and thereby upregulating tubulin. This provides a novel molecular basis for AD pathogenesis and lays a foundation for more detailed future studies [76].

#### 2.2.6 Linc01278

Wang et al. validated bioinformatic data using molecular biology approaches in AD tissues and VSMCs. Notably, Linc01278, miR‐500b‐5p, and actin gamma 2 (ACTG2) are crucial components of the VSMC contraction pathway. In AD tissues, Linc01278 and ACTG2 were downregulated, while miR‐500b‐5p was upregulated. Molecular markers of VSMC phenotypic switching—including smooth muscle 22α (SM22α), smooth muscle actin (SMA), calponin, and myosin heavy chain 11 (MYH11) – were also downregulated in AD tissues. Functional experiments showed that overexpressing Linc01278 (via plasmid) attenuated VSMC proliferation and phenotypic switching, whereas RNA interference‐mediated knockdown of Linc01278 promoted these processes. Mechanistically, dual‐luciferase reporter assays confirmed that Linc01278 sponges miR‐500b‐5p, which directly targets ACTG2, in HEK293T cells. Collectively, these data demonstrate that Linc01278 modulates VSMC phenotypic switching by regulating ACTG2 expression through the miR‐500b‐5p axis. The Linc01278/miR‐500b‐5p/ACTG2 pathway thus holds potential as a diagnostic marker and therapeutic target in AD [77].

#### 2.2.7 LncRNA PVT1

Li et al. reported that miR‐27b‐3p can competitively bind to lncRNA PVT1 in HASMCs. In AD, PVT1 expression is upregulated, whereas miR‐27b‐3p is downregulated. In PDGF‐BB–treated HASMCs, silencing PVT1 reversed the effects of PDGF‐BB on PVT1 and miR‐27b‐3p expression, as well as on phenotypic switching markers, cell viability, and migration. Conversely, inhibition of miR‐27b‐3p abrogated the effects of PVT1 silencing. Furthermore, in a mouse AD model, PVT1 knockdown alleviated the AD‐induced upregulation of PVT1, the suppression of miR‐27b‐3p, and medial degeneration of the aorta. In conclusion, PVT1 silencing suppresses the proliferation, migration, and phenotypic switching of PDGF‐BB‐treated HASMCs by targeting miR‐27b‐3p [78].

#### 2.2.8 LncRNA PTENP1

LncRNA PTENP1 is a pseudogene of the tumor suppressor PTEN and has been implicated in the regulation of VSMC proliferation and apoptosis. Lai et al. hypothesized that PTENP1 inhibits HASMC proliferation and enhances apoptosis by promoting PTEN expression. Their results showed that both PTENP1 and PTEN were upregulated in AD samples. Overexpression of PTENP1 significantly increased PTEN protein levels, promoted apoptosis, and inhibited HASMC proliferation. Conversely, silencing PTENP1 had the opposite effects and even attenuated H_2_O_2_‐induced apoptosis in HASMCs. In an Ang II‐induced mouse aortic aneurysm model, PTENP1 overexpression enhanced aortic VSMC apoptosis and exacerbated aneurysm formation. Mechanistically, RNA pull‐down assays and luciferase reporter assays (using miR‐21 mimics or inhibitors) identified PTENP1 as a sponge for miR‐21. Specifically, PTENP1 competes with PTEN mRNA for binding to miR‐21, thereby relieving miR‐21–mediated repression of PTEN and upregulating PTEN levels. This mechanism was further supported by in vitro evidence that introducing a miR‐21 mimic decreased apoptosis despite PTENP1 overexpression. Additionally, in vivo PTEN rescue experiments significantly mitigated VSMC apoptosis induced by PTENP1 overexpression. Western blot assays demonstrated that PTENP1 overexpression in HASMCs led to a substantial reduction in Akt phosphorylation, as well as decreased levels of cyclin D1 and cyclin E. Collectively, PTENP1 is identified as a key mediator of HASMC homeostasis and suggests a potential therapeutic target for intervention in AD or aortic aneurysm [79].

#### 2.2.9 LncRNA NORAD

VSMC ferroptosis is closely associated with the pathogenesis of AD. Liao et al. investigated the regulatory effects of lncRNA NORAD on VSMC ferroptosis and the underlying molecular mechanisms. Their data demonstrated that NORAD was downregulated in AD patients and in Ang II‐treated VSMCs. Overexpression of NORAD enhanced VSMC viability and inhibited Ang II‐induced ferroptosis. Mechanistically, NORAD interacted with human antigen R (HuR); this interaction increased the stability of glutathione peroxidase 4 (GPX4) mRNA, thereby elevating GPX4 protein levels. Knockdown of GPX4 abrogated the protective effects of NORAD on cell viability and ferroptosis in Ang II‐treated VSMCs. Furthermore, METTL3 catalyzed m6A methylation of NORAD in a YTHDF2‐dependent manner, promoting NORAD mRNA decay. In vivo, NORAD overexpression alleviated clinical symptoms, reduced the incidence of dissection, improved histopathological changes, and suppressed inflammation and ferroptosis in a mouse model of AD. In conclusion, METTL3‐mediated m6A modification of NORAD (via YTHDF2) leads to NORAD downregulation, whereas NORAD overexpression inhibits VSMC ferroptosis through the HuR/GPX4 axis and thereby attenuates AD progression. These findings suggest that NORAD may serve as a promising therapeutic target for AD [80].

### 2.3 Summary and Outlook

The aorta is the largest elastic artery in the human body, composed of a thin intima, a thick myoelastic media, and an outer fibrous adventitia. AD occurs when the aortic intima ruptures or hemorrhage develops within the aortic wall. As a highly fatal vascular disorder, AD has a complex pathogenesis primarily involving vascular inflammation, immune cell infiltration, and VSMC phenotypic switching and apoptosis. Among these mechanisms, VSMC phenotypic switching and apoptosis play particularly pivotal roles and are key drivers of AD development.

LncRNAs, a class of noncoding RNA molecules, exert diverse biological functions (such as acting as miRNA sponges and protein scaffolds), thereby playing essential regulatory roles in AD. Accumulating evidence indicates that in AD, lncRNAs can modulate the expression of downstream mRNAs by competitively binding to miRNAs (a regulatory paradigm known as the ceRNA network), which in turn influences AD initiation and progression. Although lncRNAs constitute approximately 80% of the human noncoding transcriptome, current AD research has focused on only a limited subset of these molecules (e.g., H19 and XIST). Moreover, most of these studies have relied on animal models or in vitro cell assays, with insufficient validation in clinical samples and a lack of in‐depth mechanistic exploration.

In summary, the pathogenesis of AD involves complex crosstalk among vascular biology, biomechanics, and immunology. As emerging regulatory factors, lncRNAs provide a novel perspective for understanding this pathological process. In silico bioinformatics analysis will serve as an important research method for screening potential lncRNA biomarkers and regulatory pathways of aortic dissection in future research, which can lay a preliminary theoretical foundation for the subsequent in‐depth verification of lncRNA functions and regulatory mechanisms in AD. Future research should prioritize the identification of lncRNAs as robust diagnostic and prognostic biomarkers for AD, the development of lncRNA‐targeted therapeutic strategies to maintain aortic wall homeostasis, and the mechanistic crosstalk between lncRNAs and major AD‐related risk factors. Further basic and translational studies will help clarify the causal roles of lncRNAs in AD and facilitate the clinical translation of lncRNA‐based precision interventions. Ultimately, these endeavors will improve early diagnosis, optimize risk stratification, and enhance effective treatment of AD, thereby alleviating the global health burden posed by this life‐threatening vascular disease.

## Author Contributions

**Qingliang Chen:** responsible for conceptualization and project management. **Zhigang Guo:** responsible for supervision, writing – review and editing. **Jie Geng:** responsible for methodology and editing. **Chao Chang:** responsible for literature curation and writing – original draft. **Yongrong Lan:** responsible for reviewing and organizing literature on the mechanism of long noncoding RNAs in aortic dissection. **Zhenhua Wu:** responsible for literature screening, framework organization, and literature formatting proofreading.

## Funding

This work was supported by Tianjin Natural Science Foundation Project, 24JCYBJC01480. Tianjin Health Research Project, TJWJ2025ZK010. Tianjin Key Medical Discipline Construction Project, TJYXZDXK‐3‐030C.

## Conflicts of Interest

The authors declare no conflicts of interest.

## Data Availability

The data used to support the findings of this study are available from the corresponding author upon request.
